# Whole Genome Sequencing of *Mycobacterium tuberculosis* Reveals Slow Growth and Low Mutation Rates during Latent Infections in Humans

**DOI:** 10.1371/journal.pone.0091024

**Published:** 2014-03-11

**Authors:** Roberto Colangeli, Vic L. Arcus, Ray T. Cursons, Ali Ruthe, Noel Karalus, Kathy Coley, Shannon D. Manning, Soyeon Kim, Emily Marchiano, David Alland

**Affiliations:** 1 Department of Medicine, Rutgers-New Jersey Medical School, Newark, New Jersey, United States of America; 2 Department of Biological Sciences, University of Waikato, Hamilton, New Zealand; 3 Department of Preventive Medicine, Rutgers-New Jersey Medical School, Newark, New Jersey, United States of America; 4 Department of Microbiology and Molecular Genetics, Michigan State University, East Lansing, Michigan, United States of America; 5 Respiratory Research Unit, Waikato Hospital, Hamilton, New Zealand; 6 Department of Pathology, Waikato Hospital, Hamilton, New Zealand; Tulane University, United States of America

## Abstract

Very little is known about the growth and mutation rates of *Mycobacterium tuberculosis* during latent infection in humans. However, studies in rhesus macaques have suggested that latent infections have mutation rates that are higher than that observed during active tuberculosis disease. Elevated mutation rates are presumed risk factors for the development of drug resistance. Therefore, the investigation of mutation rates during human latency is of high importance. We performed whole genome mutation analysis of *M. tuberculosis* isolates from a multi-decade tuberculosis outbreak of the New Zealand Rangipo strain. We used epidemiological and phylogenetic analysis to identify four cases of tuberculosis acquired from the same index case. Two of the tuberculosis cases occurred within two years of exposure and were classified as recently transmitted tuberculosis. Two other cases occurred more than 20 years after exposure and were classified as reactivation of latent *M. tuberculosis* infections. Mutation rates were compared between the two recently transmitted pairs versus the two latent pairs. Mean mutation rates assuming 20 hour generation times were 5.5X10^−10^ mutations/bp/generation for recently transmitted tuberculosis and 7.3X10^−11^ mutations/bp/generation for latent tuberculosis. Generation time versus mutation rate curves were also significantly higher for recently transmitted tuberculosis across all replication rates (p = 0.006). Assuming identical replication and mutation rates among all isolates in the final two years before disease reactivation, the u20hr mutation rate attributable to the remaining latent period was 1.6×10^−11^ mutations/bp/generation, or approximately 30 fold less than that calculated during the two years immediately before disease. Mutations attributable to oxidative stress as might be caused by bacterial exposure to the host immune system were not increased in latent infections. In conclusion, we did not find any evidence to suggest elevated mutation rates during tuberculosis latency in humans, unlike the situation in rhesus macaques.

## Introduction

Approximately one-third of the world's population is asymptomatically infected with *Mycobacterium tuberculosis* [1]. *M. tuberculosis* may persist in a latent state for the life of its human host, or may reactivate after months to years, causing symptomatic tuberculosis disease. The world's latently infected population comprises the reservoir which feeds the global tuberculosis epidemic. Therefore, novel approaches to treat and eradicate latent infections would contribute greatly to tuberculosis control and eventual eradication.

Recent immunological studies of latent tuberculosis suggest that latency encompasses a disease spectrum that extends from non-replicating persisting organisms, to replicating but asymptomatic infections, to low level-disease with higher numbers of actively replicating bacteria [2] [3]. A better understanding of the bacterial replication rates occuring during latency would help in the design of drug regimens to treat LTBI. Replicating organisms are susceptible to drugs that target pathways required for cellular growth. Several commonly used anti-tuberculosis drugs fall into this category. On the other hand, non-replicating organisms are much more tolerant to these drug classes [4] [5]. Another unknown feature of latency is the mutational capacity of the latent *M. tuberculosis* bacilli. The presumed intracellular location and prolonged exposure of latently infecting *M. tuberculosis* to reactive effecter molecules of the host immune system could predispose latent organisms to enhanced rates of mutation [6]. Rapidly mutating organisms are more likely to undergo selection for drug resistance. Thus, our understanding of replication and mutagenesis in latent *M. tuberculosis* has clear implications for treatment strategies to eradicate latency.

The replication and mutation rates which occur during latency are difficult to individually verify experimentally. However, a curve which describes mutation rates as a function of replication (expressed in generation times) can be calculated by measuring the number of chromosomal mutations that occur over a defined period of infection [6]. Recent studies in rhesus macaques have shown that the generation-time versus mutation-rate (GT vs MR) curves of *M. tuberculosis* isolated from latent lesions and reactivated infection are surprisingly similar to those of *M. tuberculosis* isolated from active pulmonary disease [6]. These observations suggest either that latent and disease-causing *M. tuberculosis* have strikingly similar replication and mutation rates, or that latent *M. tuberculosis* has lower replication rates than disease-causing *M. tuberculosis*, but has substantially higher mutation rates.

We considered the possibility that the non-human primate model might not appropriately recapitulate latent tuberculosis in humans. Latent tuberculosis cannot be isolated from living humans. However, GT vs MR curves during latency may be inferred from the genome sequence of strains isolated from subjects who develop tuberculosis disease after a long latency period in comparison to the genome sequence of the strain isolated from the original index case. A similar analysis of strains isolated from recently transmitted tuberculosis cases can serve as a comparator group. This type of analysis must be performed using strains from a well defined tuberculosis outbreak caused by an easily identifiable clone, preferably in a setting where the background tuberculosis infection rate with confounding strains is low. The multi-decade outbreak of the *M. tuberculosis* Rangipo strain in New Zealand provided us with such an opportunity to study *M. tuberculosis* replication and mutation rates in tuberculosis disease caused by recently transmitted *M. tuberculosis* versus disease that developed after a long latency period [7]. This investigation describes the first study of latent *M. tuberculosis* bacteria in humans.

## Results

### Epidemiology of outbreak

Over the past several decades, an *M. tuberculosis* strain locally known as the Rangipo strain has been responsible for a number of tuberculosis outbreaks in New Zealand. Tuberculosis incidence rates in the central North Island have been constant over this period (1991–2011) at ∼8 per 100,000, however outbreaks of the Rangipo strain have resulted in local spikes in incidence rates. For example, an outbreak of this strain in the Hawkes Bay region increased incidence rates from a baseline of 8.7 per 100,000 before 1988 to a rate of 20.9 per 100,000 for the period between 1988 and 2002. The Rangipo strain has been typed using IS6110-based DNA fingerprinting and is the largest single *M. tuberculosis* cluster in New Zealand representing over 12% of cluster cases (57 cases over 5 year period). [8] This strain is significantly more likely to infect people of Maori or Pacific ethnicity and people under the age of 20 years.

**Figure 1:**
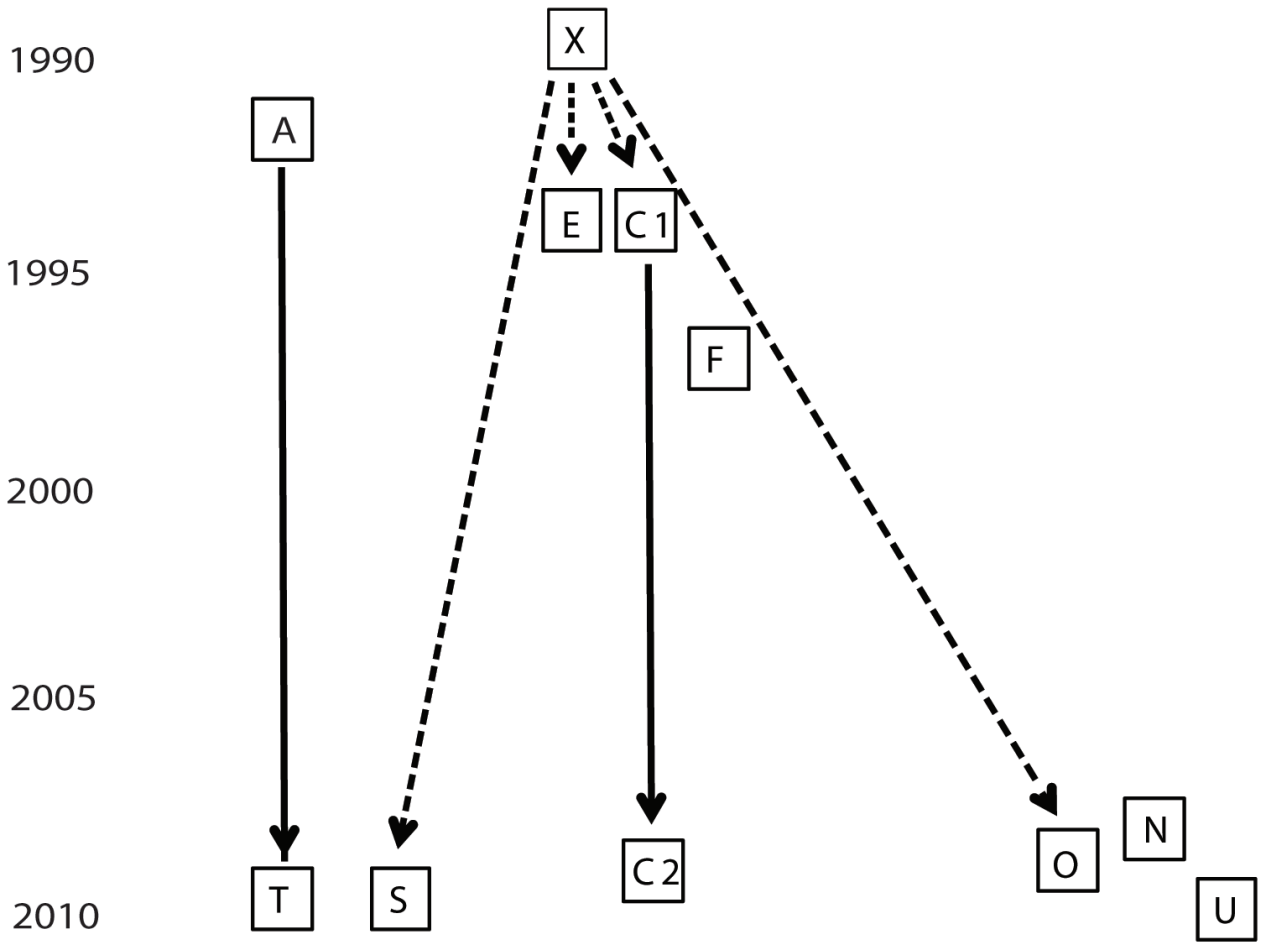
Epidemiological relationships among 11 New Zealand *M. tuberculosis* cases. Chronological representation of different subjects in a New Zealand TB outbreak. Each square represent a subject at the time of the TB diagnosis. Broken lines represent known close direct contact with the initial index case “X” during X's period of infectiousness. Solid lines show assumed connections between a case of presumed reactivation (C1 to C2) and a case of potential child-parent transmission (A to T).

We investigated a single-source Rangipo strain cluster plus same-strain controls (Fig. 1) that occurred over a 20 year period in central North Island of New Zealand. Subject “X” developed Rangipo-strain tuberculosis in 1990 and appears to the index cases for the cluster of interest. The sibling and close contact of X, subject “C1” was subsequently diagnosed with culture-positive tuberculosis in 1992. Subject C1 was treated and appeared to be cured of tuberculosis, but the subject again developed tuberculosis 17 years later in 2009 (now called subject “C2”). C2 had also developed diabetes by this time. Subject ”E” was a close contact of subject X, who developed tuberculosis meningitis in 1992. Subjects “S” and “O” were also close contacts with subject “X” in the early 1990's. S developed tuberculosis in 2010 and O developed tuberculosis in 2008. For control cultures, we selected other tuberculosis cases caused by the Rangipo-strain in the same region of New Zealand from subjects who did not have any known contacts with subject X, or with subjects E, C1/C2, or, S. Subject “A” had tuberculosis in 1991 at the age of 35. Subject “T” was the parent of subject “A”. T had close contact with A during the time that A had active tuberculosis in the 1990's; however, T did not develop tuberculosis until in 2010. Subject “F” was a 3 year old child who had tuberculosis in 1996. Subjects “N”, “O”, and “U” developed tuberculosis in 2007, 2008 and 2011, respectively.

### Identification of polymorphisms among *M. tuberculosis* NZ isolates

We sequenced the complete genomes of all 10 outbreak and control *M. tuberculosis* strains to establish their phylogenetic relationships and to estimate the replication and mutation rates of various isolate pairs. Paired isolates X – E, X- C1 were assumed to represent recently-transmitted tuberculosis (RT-TB) with subsequent progression or early-reactivation to disease because E and C1 developed tuberculosis within two years of exposure to X's tuberculosis. X – S, X – O, C1 – C2 and A – T pairs were assumed to represent latent tuberculosis (L-TB) with late-reactivation to disease because S, O, and T developed tuberculosis >10 years after exposure to their index case, and C2 developed a second case of tuberculosis 17 year after apparently successful treatment for this subject's (C1) first tuberculosis episode.

Genome coverage averaged 98% across these isolates, and the average read depth was 120X across the genomes (Table S1). A total of 747 SNPs were found between the H37Rv strain reference genome and the study isolates. After eliminating SNP differences with H37Rv that were shared by all 10 sequenced isolates, SNPs in repetitive sequences, and errors in SNP calls discovered by our Sanger re-sequencing efforts, we identified a total of 34 SNPs in the 10 sequenced genomes. In no case did we find a confirmed SNP between two colonies isolated from the same subject sample. Thus, all subsequent analyses were performed on a per-subject basis (Table 1). The isolates from each subject differed from each other subject isolate by a minimum of 1 SNPs and a maximum of 14 SNPs (Table 2). We examined the types of genes which were mutated between our study subjects. The mutations did not cluster into any particular gene or functional type. SNPs were identified in genes belong to secreted antigen (3/34); hypothetical proteins (6/34); membranes associated and transport proteins (4/34); transcriptional regulators (5/34); enzymes for degradation or biosynthesis (10/34); proteases (2/34); and factors involved in translation (2/34).

**Table 1:** Single nucleotide polymorphisms identified in sequenced isolates.

	Strain										
	A	C1	E	F	N	O	C2	S	T	U	H37Rv^1^
Gene											
Rv3705c	-	-	-	-	-	-	A	-	-	-	G
Rv3519	-	-	-	-	-	-	A	-	-	-	T
Rv3555c	-	-	-	-	-	-	-	-	C	-	G
Rv1081c	-	-	-	-	A	-	-	-	-	-	C
*mutA*	-	-	-	-	-	-	-	-	G	-	A
Rv0024	-	-	-	-	C	-	-	-	-	-	A
Rv2333c	-	A	-	-	-	-	-	-	-	-	G
Rv0047c	-	-	-	-	-	-	-	-	A	-	G
Rv0338c	-	-	-	-	-	-	T	-	-	-	C
Rv0241c	-	-	-	-	-	A	-	-	-	-	C
*ctpF*	-	A	-	-	-	-	-	-	-	-	G
*adhE1*	-	-	-	-	-	-	-	-	T	-	C
Rv2689c	-	-	-	-	-	-	T	-	-	-	C
*bioF2*	-	-	-	-	-	-	-	-	C	-	G
*esxO*	-	-	-	-	A	-	-	-	-	-	G
*ppsD*	G	-	-	-	-	-	-	-	-	-	T
*clpX*	-	-	-	-	-	C	-	-	-	-	T
*fadD34*	-	-	-	-	C	-	-	-	-	-	G
Rv2282c	-	-	-	-	-	-	-	-	-	G	A
*oxyS*	-	T	-	-	-	-	-	-	-	-	G
*nirB*	-	-	-	-	-	-	-	-	-	T	C
*infB*	-	-	-	-	-	-	-	-	-	G	T
Rv1626	-	-	-	-	-	-	-	-	T	-	C
*valS*	-	-	-	-	T	-	-	-	-	-	G
Rv1231c	-	-	-	-	-	-	-	-	T	-	C
Rv3559c	-	-	-	-	-	-	-	-	G	-	C
*glnA2*	-	-	-	-	-	-	-	-	-	G	A
Rv0737	-	-	-	A	-	-	-	-	-	-	C
*clpB*	-	-	-	A	A	-	-	-	-	-	G
Rv3586	-	-	-	A	A	-	-	-	-	-	G
Rv1422	-	-	T	-	-	-	-	-	-	-	C
*lldD2*	T	-	-	-	T	T	T	T	T	T	C
*fadE21*	G	-	-	-	G	G	G	G	G	G	A
Rv3213c	-	-	-	-	-	-	-	-	A	-	G

1 reference genome.

**Table 2:** Number of single nucleotide polymorphisms differences between each strain.

	Strain X	Strain C1	Strain E	Strain S	Strain A	Strain F	Strain N	Strain O	Strain C2	Strain T
Strain X	0									
Strain C1	3	0								
Strain E	1	4	0							
Strain S	2	3	1	0						
Strain A	3	4	2	1	0					
Strain F	3	4	2	1	2	0				
Strain N	9	8	6	5	6	6	0			
Strain O	4	5	3	2	3	3	7	0		
Strain C2	6	7	5	4	5	5	9	6	0	
Strain T	11	12	10	9	10	10	14	11	13	0
Strain U	6	6	4	3	4	4	8	5	7	12

### Phylogenetic analysis

**Figure 2:**
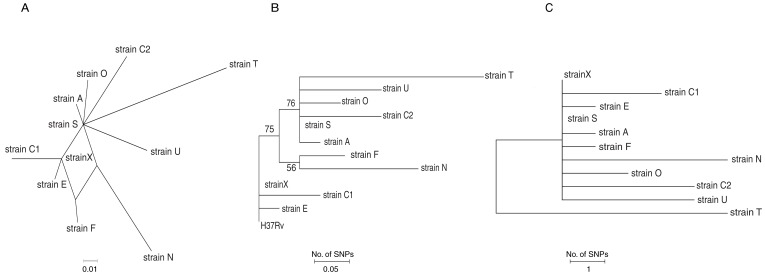
Evolutionary history of *M. tuberculosis* strains isolated from a cluster of cases in New Zealand. Relationships were inferred by examining parsimony and nonparsimony informative single nucleotide polymorphisms (SNPs). **Panel A**: parallelogram of network analysis using 34 SNPs including five homoplastic SNPs. **Panel B**: Evolutionary tree using the same 34 SNPs as in A rooted on *M. tuberculosis* H_37_Rv analyzed using Neighbor-joining method. Panel C. Evolutionary tree examining 29 nonparsimony SNPs using the Neighbor-joining method. The evolutionary distances represent the number of SNP differences per strain.

A phylogenetic analysis of all 34 SNPs from the 10 Rangipo genomes was performed. Five of the SNPs appeared to be homoplastic, as indicated by the parallelograms shown in a network analysis (Fig. 2A); however, a pairwise homoplasy index test for recombination could not be performed due to the small number of SNPs evaluated. Homoplasy suggests either convergent evolution of independent mutational events or lateral gene exchange (although gene exchange appears to be uncommon in *M. tuberculosis*) [9]. The 34 SNP phylogenetic analysis supported the epidemiological connections identified between subjects X - E, X - C1, and X - S. However, a direct connection between X and O was somewhat less certain given that S and O shared one SNP each in *fadE*21 and *ild*2 that were not present in X (Table 1). Surprisingly, the phylogenetic analysis suggested that C2 did not have C1 as its most recent ancestor even though subject C2 was in fact the same person as subject C1. Similarly, isolate T was confirmed to not have isolate A as its most recent ancestor, even though we had originally assumed that subject T had contracted tuberculosis from his/her child, subject A. Similar phylogenetic relationships were observed in a 34 SNP analysis using the Neighbor-joining method with rooting on *M. tuberculosis* strain H37Rv (Fig. 2B).

Homoplasy can interfere with an accurate phylogenetic analysis [10]. Therefore, we reasoned that a more accurate description of isolate relationships and ancestry would be obtained by performing a phylogenetic analysis that included only the 29 non-parsimonious SNPs using the Neighbor-joining method (Fig. 2C). In this analysis, all isolates except that from subject T shared a common root, and X was the most deeply rooted subject. This analysis further confirmed the close ancestry of the X – E, X – C1, and X – S strain pairs, and strongly supported the epidemiologically suggested ancestry of the X – O strain pair. As in the 34 SNP analyses, the 29 SNP analysis also indicated that isolate C2 did not have isolate C1 as its most recent ancestor; and isolate T did not have A as its most recent ancestor. These results demonstrate that epidemiological connections such as presumed relapse, or close contact between a child and parent do not conclusively prove ancestry in an outbreak investigation, even if the isolates have matched DNA fingerprints.

We looked for additional ways to test whether O and S were caused by reactivation of a latent infection acquired from X around 1990, or were instead acquired more recently from Rangipo strains circulating in the community during the past decade. We reasoned that reactivated latent infections should be similar to other strains such as E and C1, isolated from contacts with X in the 1990s as measured by SNP distance. In contrast, more recently acquired infections should be more similar to stains circulating in the past decade, such as T, C2 N and U. Indeed, the mean SNP distance (Table 2) among O, S, E and C1 was 2.8, while the mean SNP distance among O, S, T, C2, N and U was 6.4, with a two sided p-value of 0.057. While the difference between these two groups did not quite reach statistical significance, the trend for significant difference was quite strong. These results further support our conclusion that S and O derive from reactivation of a latent X infection instead of infection from a more recently circulating Rangipo strain.

### Mutation rates among *M. tuberculosis* isolates

**Figure 3:**
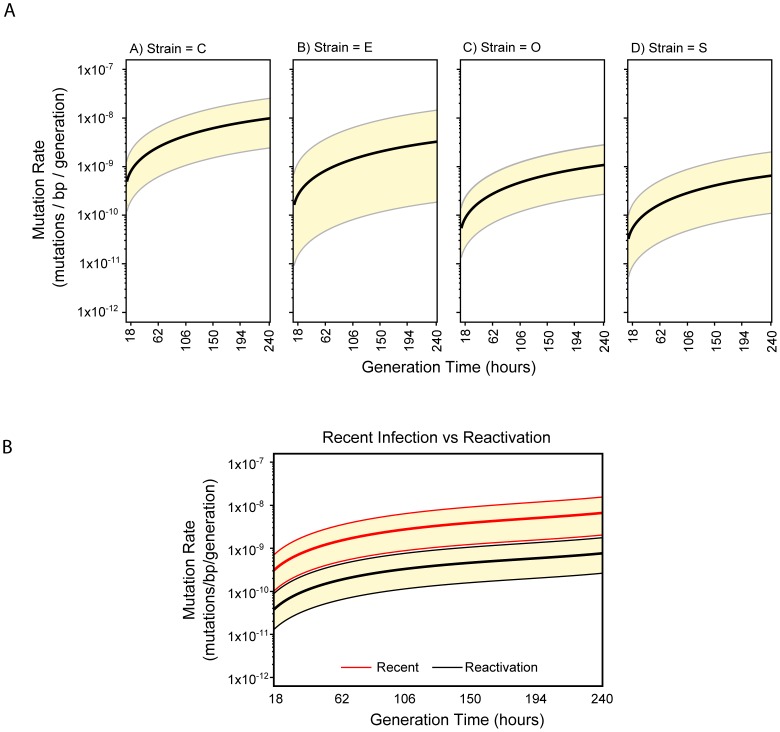
In vivo mutation rates in *M. tuberculosis* strains for generation times ranging from 18 to 240 hours. The mutation rate for each of the *M. tuberculosis* isolates was estimated using equation (1) in Ford *et al*, and calculated as untis of mutation/bp/generation **Panel A**: The mutation rate for each *M. tuberculosis* isolate in the study. Isolate C1 and E are from recent infection while strains O and S from reactivation after a prolonged period of latency. The yellow areas represent 95% confidence intervals. **Panel B**: Isolates with recent infection (E and C1) and reactivation after prolonged latency (O and S) were combined to obtain overall estimates of mutation rates of recent and latent-reactivated disease. The yellow areas represent 95% confidence intervals.

We examined the mutation rates for each isolate pair; after elimination pairs A–T and C1–C2 due to the lack of phylogenetic support for direct ancestry. We estimated the mutation rate mutation/bp/generation across a broad range of generation times, and also derived a mutation rate at an assumed 20 hour generation time (u20hr) as described previously [6]. Our results differed strikingly from those derived from rhesus macaques. The estimated u20hr mutation rate for the RT-TB isolate pairs X-C1 and X-E was 8.2×10^−10^ and 2.7×10^−10^ mutations/bp/generation respectively, while for the L-TB isolate pairs X-S and X-O, the u20h was 5.4×10^−11^ and 9.1×10^−11^ mutations/bp/generation. The GT vs MR curves of the RT-TB isolate pairs also differed markedly compared to the L-TB isolate pairs (Fig 3A). If a common generation time is assumed, the estimated mean mutation rates per generation or alternatively the mean number of mutations per calendar year of the RT-TB isolate pairs was approximately 10-fold higher than those of the L-TB isolate pairs (Fig 3B), with a significant difference of p = 0.006 between the two conditions.

**Figure 4:**
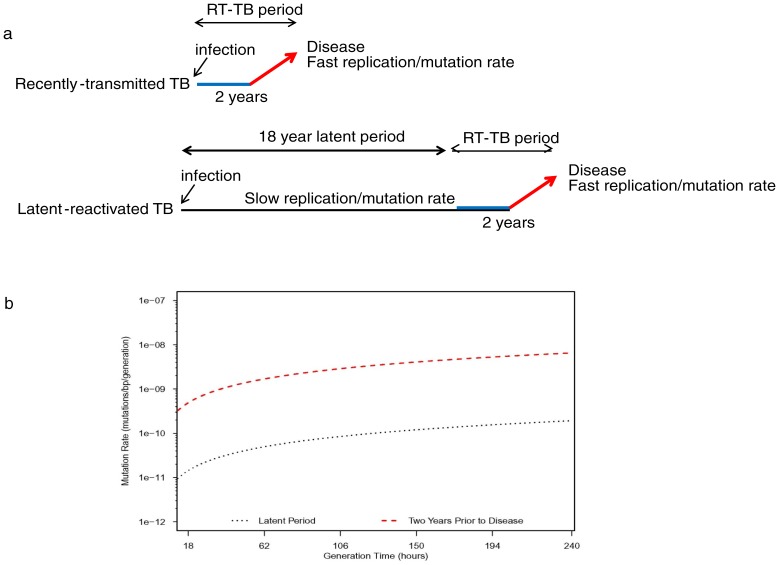
Mutation versus replication rates during the latency period. To evaluate if the mutation rate was different in early versus late period of latent-reactivated disease, we estimated the mutation rate (calculated as untis of mutation/bp/generation) for isolates with reactivation of TB for the latent and reactivated years. **Panel A**: A schematic representation for the evaluation of the rate of mutation in the latency period. The mutation rate of the recent activated TB (strains C1 and E) was subtracted from the late activated TB (strains O and S).**Panel B**: The mutation rate for the latent period was calculated using the difference between the number of mutations for the early reactivation phase and the late reactivated phase. The result was divided by the genome size multiplied by the number of generations for O and S combined in the presumed latent period. All these calculations implicitly assume a homogeneous mutation rate across the genome and over time and when combining activation groups, across isolates. Analyses were performed using PROC GENMOD in SAS 9.2 (SAS Institute Inc, Cary, NC). Graphics were generated using R 2.12.2 (R Foundation for Statistical Computing).

Even though the L-TB isolates experienced a long period of latency, they ultimately reactivated to cause clinical tuberculosis. Thus, a period of the L-TB isolate lifecycle may have been similar to that of the RT-TB isolates including replication and mutation rates similar to the RT-TB isolates. We wished to specifically study the mutation versus replication rates of the latency period. For these calculations, we made the assumption that the RT-TB and L-TB isolate groups have identical replication and mutation rates in the two years preceding the onset of each subject's clinical disease (Fig. 4A). Adjusting for this two-year period, we calculated the u20hr mutation rate attributable to the remaining latent period to be 1.6×10^−11^ mutations/bp/generation, or approximately 30 fold less than that calculated during the final two years before disease. The GT vs MR curves were similarly depressed (Fig 4B). In fact, we found that the mutation rate during the modeled latent period at a generation time of 240 hours (the end of our scale) was lower than the mutation rate at a generation time of 18 hours (the beginning of our scale) during the modeled two years before disease period. These results strongly suggest that replication rates and/or mutation rates are markedly lower during the latent stage of disease compared to the two-year period up to and including active tuberculosis.

**Figure 5:**
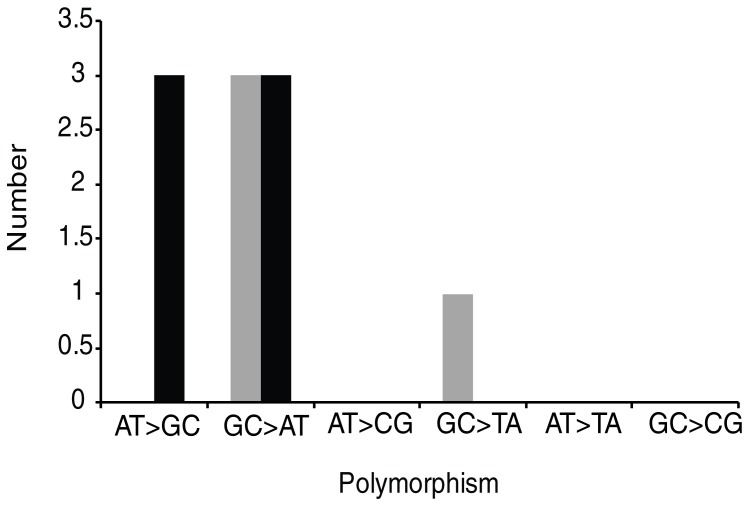
Different type of mutations among recent and latent reactivated *M. tuberculosis* isolates. Isolates from subjects with recent (Gray) and latent (Black) reactivated tuberculosis were analyzed for different mutations types. GC>AT and GC>TA mutations represent changes potentially due to oxidative damage.

Finally, we compared the type of mutations which developed in the RT-TB versus the L-TB groups to determine the degree to which oxidative damage could account for mutation rates. We looked for footprints of oxidative damage reflected by either cytosine deamination (GC to AT changes), or the formation of 8-oxoguanine (GC to TA changes) [11] [12]. A total of 10 SNPs were detected among the four isolate pairs. All four of the mutations present in the RT-TB group were indicative of oxidative damage (Fig 5). In contrast only three of six mutations in the L-TB group were of this type. Thus, half of the mutations in the L-TB group can be attributed to replicative mutagenesis rather than oxidative stress. It is not possible to know which mutations occurred during the prolonged latency period versus the accelerated replication rates that we presume to have occurred just prior to and during active disease. However, our results do not suggest a higher rate of oxidative-damage induced mutagenesis during the latent phase compared to the phase of active tuberculosis in humans, which contrasts strongly with rhesus macaques.

## Discussion

It is understandably difficult to investigate the biology of the *M. tuberculosis* during latent human infections. *M. tuberculosis* can cause years of asymptomatic infection, only to reactivate when the host immune system is perturbed, or by other unknown causes. It is difficult or impossible to culture *M. tuberculosis* from these asymptomatic individuals until they develop symptomatic disease. Latent tuberculosis is instead detected by way of the host immune response. We were able to estimate the replication and mutation rate of latent *M. tuberculosis* bacilli by comparing the genome sequence of a single strain that had been transmitted from a single index case, causing disease in close contacts over a period of >20 years. We found that mutation rates were substantially lower during latency for any given generation time, especially after we adjusted for the higher mutation rates that were likely to have occurred during the final phase of each subject's infection, as they developed active tuberculosis.

Our study did not enable us to separately calculate replication and mutation rates for the conditions under investigation. Instead, each parameter could only be considered as a function of the other. However, latency is defined by an absence of symptoms, which suggests that replication rates are likely to be lower during latency than they are during active clinical tuberculosis. Assuming that this is true, our results are best explained by a picture of latency that involves low replication rates and low mutation rates compared to active tuberculosis. Our conclusions are further supported by the fact that mutations attributable to oxidative stress were not found in a higher proportion of the L-TB isolate pairs compared to the RT-TB pairs, rather the opposite was true. This observation also contrasts to the situation in rhesus macaques. Although our analysis was relative small (based on four pair-wise comparisons), it is comparable to the sample size used in the rhesus macaques studies were four animals were used for active disease, three for latent and two for reactivation. Furthermore, although different *M. tuberculosis* lineages have been found to have different intrinsic mutation rates during in vitro culture [6], the Rangipo strain is a member of lineage 4, which is the same group as the Erdman and H_37_Rv strain used in the macaque study. In addition, the close SNP-defined relationship among all of the study isolates strongly suggests that our results are not due to differences in intrinsic mutation rates between L-TB and RT-TB isolates. Thus, it appears that human latency may differ markedly from latency in non-human primates. Human latency appears to lie on the quiescent end of the disease spectrum while latency in rhesus macaques appears to be closer to active disease. We were only able to study two subjects with long periods of latent infection; therefore, it is certainly possible that a much broader spectrum of replication and mutation rates exists among the billions of humans with latent tuberculosis infection. Our results will need to be validated in larger studies, with different patient populations and *M. tuberculosis* strain types.

We did not find evidence that latent *M. tuberculosis* bacilli are under excessive oxidative mutational stress during latency, suggesting that mutational drug resistance is unlikely to occur during a latent infection, especially if the bacterial burden is low. It follows that latent infection is likely to be safely treatable with single drugs, as is common practice. The risk for mutational drug resistance would be expected to rise as a latent infection begins to progress to active tuberculosis disease, however. Thus, multi-drug therapy might be considered to prevent resistance from developing during latent infections that are deemed to be at high risk for rapid reactivitation, as might be the case after a known recent exposure to a tuberculosis case. Immunological assays of host response or more sensitive diagnostic assays to detect rare *M. tuberculosis* organisms may be needed to distinguish between the latent and early disease states.

Our results help to confirm the hypothesis that improved drugs targeting latent *M. tuberculosis* might be able to work through novel mechanisms which kill non-replicating or slowly replicating organisms. Several new anti-tuberculosis drugs under development may be suitable and should be studied for this purpose. On the other hand, our results suggest that replication does take place in latent *M. tuberculosis*, infection, albeit at a slow rate. These findings help to explain how drugs such as isoniazid, which only kill replicating bacteria, can effectively eradicate latent infections when treatment is extended for nine months.

Our study is consistent with and expands on other whole genome sequencing studies of clinical *M. tuberculosis* isolates. Sandegren et al. showed that the *M. tuberculosis* genome is quite stable in a serial chain of transmission across 115 subjects [13]. Others have described the utility of whole genome sequencing in defining tuberculosis outbreaks [14] [15]. As in our study, Namouchi et al. [16] detected homoplastic SNPs on the terminal branches of an *M. tuberculosis* phylogenetic tree, suggesting that convergent evolution or lateral gene exchange can occur. Our study also provides a number of new insights into the use of common molecular epidemiological methods and tools. Classical epidemiology and molecular fingerprinting had suggested the isolate C2 could be attributed to relapse tuberculosis in subject C1. In a similar manner, subject T appeared to have acquired tuberculosis from subject A. Our phylogenetic analysis suggests that neither of these strong epidemiologic connections explained the origin of these two cases. As powerful as whole genome sequencing may be for establishing transmission links and strain identification, our results emphasize the importance of including same-strain controls and performing detailed phylogenetic analyses of all samples.

In summary, this study provides the first estimates of replication and mutation rates in latent human tuberculosis, and strongly supports the concept that latent tuberculosis in humans differs from that documented in rhesus macaques. Our results suggest that human latency can include prolonged periods with very low replication rates and only moderate rates of mutations. These results demonstrate the important contributions and limitations of whole genome sequencing in the study of latency. Further investigations will be required to fully elucidate the biological features of this disease state.

## Methods

### Bacteria culture and DNA isolation

*M. tuberculosis* isolates from New Zealand were collected as follows: Clinical samples were cultured on Lowenstein Jensen (LJ) slopes, and in Bactec 12B medium, a modified Middlebrook broth supplemented with OADC enrichment and PANTA antibiotic mixture. Isolates collected in the late 1990's were cultured on LJ slopes and Mycobacteria Growth Index Tubes (MGIT). After isolation, cultures were stored in Middlebrook 7H9 broth/MGIT broth at −70°C. *M. tuberculosis* from these New Zealand cultures were then subcultured at 37 C on Middlebrook 7H10 media (Difco, Detroit, Mich.) containing 0.05% Tween 80, 0.02% glycerol, and 10% OADC. Two individual colonies from each original culture were selected at random, cultured to mid log phase in Middlebrook 7H9 media containing 0.05% Tween 80, 0.02% glycerol, and 10% OADC then plated onto 7H10 media to obtain confluent growth. After substantial colony growth was observed, all of the colonies on each plate were scraped into water and then DNA from these pooled colonies was isolated using a hexadecyltrimethylammonium bromide (CTAB) extraction protocol as described previously [17]. All the *M. tuberculosis* isolates were part of a single outbreak of the Rangipo strain [8] with a MIRU pattern for completeness of 233 325 153 324 341 444 223 362.

### Genome Sequencing

Barcoded libraries were prepared by shearing genomic DNA to 100 to 400 according to the manufacturer's recommendation (Applied Biosystems (ABI), Foster City, CA P/N 4443713). Each library was ligated to the prepared DNA and the libraries were nick translated and amplified using multiplex library PCR primers. After multiple purification steps the DNA was mounted on the SOLiD 5500XL instrument (ABI). Data was collected for each cycle in the Instrument Control Software (ICS) and transferred to the server where analysis is performed in the SOLiD Experimental Tracking Software (SETS). The overall fold coverage for each *M. tuberculosis* strain ranged between 392 and 40, while the fraction covered by the sequence ranged from 0.96 to 1.00 (Table S1). The pipeline analysis was performed using CLC Genomics Workbench (CLCbio, Denmark). After uploading the data files to the CLC server the sequence reads were trimmed to remove short and low quality reads (length >20 bases, quality≥0.05) and mapped to the Mtb genome (NCBI accession #N000962) [18]. All statistical information was derived from the resulting table generated by the software. Sequences with multiple alignments to the reference strain were removed, these sequences principally corresponded to the highly repetitive PE/PPE, PE-PGRS gene families and mobile genetic elements. We also removed alignments with<10X coverage.

### Mutation detection and confirmation

After trimming, CLC Genomics Workbench was used to identify single nucleotide polymorphisms (SNPs) in the subject sequences compared to the H37Rv genome. SNPs were discarded if <80 of the reads showed this polymorphism. All of the loci that were mutant in at least one study isolate were concatenated for each sequenced genome. However, *M. tuberculosis* cultures were not available for subject X, therefore this subject's concatenated sequence was created using the consensus of the subject E and C (close contacts of subject X) concatenations. The subject S concatenation was used to break a tie when subject E and C differed. Mutations found to be common to all of the study isolates compared to the reference strain were eliminated. Mutations unique to one subject but present in both colonies from that subject (concordant paired mutations) were accepted without further evaluation after an initial Sanger sequencing test of four concordant pair mutations confirmed sequence accuracy in this situation). Discordant paired mutations were analyzed using CLC Genomics Workbench and mutations which were near the predefined cutoffs were confirmed by Sanger sequencing of both paired isolates. DNA sequencing primers are listed in Table S2.

### Phylogenetic analysis

A total of 34 SNPs representing the 10 strains were concatenated and aligned in MEGA 5.05 [19] and the evolutionary history was inferred using the Neighbor-Joining method [20] with bootstrap confidence values (1000 replications) [21]. The evolutionary distances were computed using the p-distance method [22] and are represented as the number of base distances per site. Additionally, a phylogenetic network was constructed using the Neighbor-net algorithm [23], which was applied to 30 parsimonious informative sites using SplitsTree4 [24].Trees were constructed with and without Rv as an outgroup.

### Statistical analysis and estimation of mutation rate

As in Ford *et al*, [6] the mutation rate for each strain was estimated from the number of SNPs observed divided by the number of generations, where the number of generations was calculated from the time to (re)activation divided by the generation time which was varied between 18 and 240 hours. A Poisson model was assumed for the number of SNPs with offsets used to normalize the rates to the (re)activation time. The 95% confidence intervals using the profile likelihood were calculated on the log scale and transformed to the original scale. Analyses were performed using PROC GENMOD in SAS 9.2 (SAS Institute Inc, Cary, NC). Graphics were generated using R 2.12.2 (R Foundation for Statistical Computing). To test whether strains epidemiologically linked to X had shorter SNP distances within the group compared to those occurring between 2000–2011 and were not linked to X, we generated all permutations of four cases and generated the average SNP distance within the group by generating all pairs within the groups of 4. We compared the average SNP distance for the observed epidemiologically linked group (S, O, E, C1) to the permutation distribution to obtain an exact two-sided p-value.

## Supporting Information

Table S1 (DOCX)

Table S2 (XLSX)
