# Supplementary material for: Whole Genome Sequencing of Mycobacterium tuberculosis Reveals Slow Growth and Low Mutation Rates during Latent Infections in Humans
Source: PLoS One. 2014 Mar 11;9(3):e91024. doi: 10.1371/journal.pone.0091024 (PMC3949705; doi:10.1371/journal.pone.0091024)
Supplement: Table S1 — (DOCX) [file pone.0091024.s001.docx]

Table S1

| **Sample** | **total reads** | **mapped reads** | **fold coverage** | **fraction covered** |
| --- | --- | --- | --- | --- |
| A | 1.55E+07 | 9.57E+06 | 99 | 0.99 |
| A1 | 2.93E+07 | 1.78E+07 | 150 | 0.96 |
| C1-1 | 2.49E+07 | 6.13E+06 | 84 | 0.97 |
| C1-2 | 1.90E+07 | 1.24E+07 | 135 | 1.00 |
| E | 2.19E+07 | 1.64E+07 | 149 | 0.99 |
| E1 | 6.04E+07 | 4.54E+07 | 392 | 1.00 |
| F | 2.97E+07 | 1.86E+07 | 203 | 0.98 |
| F1 | 2.21E+07 | 1.59E+07 | 155 | 0.97 |
| N | 1.35E+07 | 4.61E+06 | 58 | 0.96 |
| N1 | 1.07E+07 | 4.16E+06 | 59 | 0.98 |
| O | 5.72E+07 | 3.84E+07 | 205 | 0.98 |
| O1 | 2.48E+07 | 1.49E+07 | 160 | 0.97 |
| C2-1 | 8.86E+06 | 3.64E+06 | 50 | 0.97 |
| C2-2 | 1.65E+07 | 2.53E+06 | 52 | 0.97 |
| S | 1.34E+07 | 6.50E+06 | 40 | 0.95 |
| S1 | 1.36E+07 | 6.50E+06 | 40 | 0.97 |
| T | 1.38E+07 | 1.08E+07 | 97 | 0.98 |
| T1 | 1.50E+07 | 1.21E+07 | 107 | 0.98 |
| U | 6.92E+07 | 5.55E+06 | 49 | 0.98 |
| U1 | 1.68E+07 | 1.24E+07 | 107 | 0.98 |
